# Mesenchymal progenitor cell markers in human articular cartilage: normal distribution and changes in osteoarthritis

**DOI:** 10.1186/ar2719

**Published:** 2009-06-05

**Authors:** Shawn P Grogan, Shigeru Miyaki, Hiroshi Asahara, Darryl D D'Lima, Martin K Lotz

**Affiliations:** 1 Department of Molecular and Experimental Medicine, The Scripps Research Institute, 10550 North Torrey Pines Road, La Jolla, California, 92037, USA; 2 Shiley Center for Orthopaedic Research and Education at Scripps Clinic, 11025 North Torrey Pines Road, Suite 140, La Jolla, California, 92037, USA

## Abstract

**Introduction:**

Recent findings suggest that articular cartilage contains mesenchymal progenitor cells. The aim of this study was to examine the distribution of stem cell markers (Notch-1, Stro-1 and VCAM-1) and of molecules that modulate progenitor differentiation (Notch-1 and Sox9) in normal adult human articular cartilage and in osteoarthritis (OA) cartilage.

**Methods:**

Expression of the markers was analyzed by immunohistochemistry (IHC) and flow cytometry. Hoechst 33342 dye was used to identify and sort the cartilage side population (SP). Multilineage differentiation assays including chondrogenesis, osteogenesis and adipogenesis were performed on SP and non-SP (NSP) cells.

**Results:**

A surprisingly high number (>45%) of cells were positive for Notch-1, Stro-1 and VCAM-1 throughout normal cartilage. Expression of these markers was higher in the superficial zone (SZ) of normal cartilage as compared to the middle zone (MZ) and deep zone (DZ). Non-fibrillated OA cartilage SZ showed reduced Notch-1 and Sox9 staining frequency, while Notch-1, Stro-1 and VCAM-1 positive cells were increased in the MZ. Most cells in OA clusters were positive for each molecule tested. The frequency of SP cells in cartilage was 0.14 ± 0.05% and no difference was found between normal and OA. SP cells displayed chondrogenic and osteogenic but not adipogenic differentiation potential.

**Conclusions:**

These results show a surprisingly high number of cells that express putative progenitor cell markers in human cartilage. In contrast, the percentage of SP cells is much lower and within the range of expected stem cell frequency. Thus, markers such as Notch-1, Stro-1 or VCAM-1 may not be useful to identify progenitors in cartilage. Instead, their increased expression in OA cartilage implicates involvement in the abnormal cell activation and differentiation process characteristic of OA.

## Introduction

The limited repair capacity of adult articular cartilage represents one factor involved in the development of progressive cartilage degeneration and osteoarthritis (OA) following cartilage injury. This notion was previously related to the absence of an inflammatory response, the putative absence and lack of access to stem cells in cartilage [1,2], and intrinsic limitations of adult human articular chondrocytes (AHAC) to repair tissue damage [3]. Yet, when cultured under appropriate conditions, cells isolated from cartilage can be induced to form cartilage-like tissue *in vitro *[4] and monolayer-expanded AHAC can form hyaline-like tissue when implanted into cartilage defects *in vivo *[5].

Cells in OA cartilage are activated as evidenced by the increased expression of a large number of genes and certain cells proliferate to form the characteristic cell clusters [6,7]. This cell activation is also associated with abnormal cell differentiation and represents a central pathogenetic mechanism in OA [6-9]. Recent studies suggest the presence of cells that express mesenchymal stem cell (MSC) markers and possess multilineage differentiation capacity in normal articular cartilage [10-12]. A new interpretation of the cellular responses in OA tissue is the possible involvement of resident cartilage progenitor cells [13] and is consistent with our previous report of increased progenitor marker expression in OA cartilage [14].

Although much information is available on the potential use of MSC in tissue engineering [15], the functions of these cells in tissue homeostasis and in arthritis pathogenesis are largely unknown. MSC can be isolated from various tissue sources but most of the current knowledge on MSC biology is based on studies with bone marrow-derived MSC (BM-MSC) [16]. These cells have the capacity to form various mesenchymal tissues such as bone, adipose tissue, tendon, muscle, and cartilage [17,18]. BM-MSC have been characterized by the expression of several cell surface antigens [19-23]. Despite the identification of these candidate markers there is, at present, no consensus on a single marker for MSC [24]. Combinations of cell surface molecules are often employed to identify progenitor cells [20] and include Stro-1 [23,25], CD105/endoglin (transforming growth factor (TGF) β receptor III) [25], CD73 (an ecto-5'-nucleotidase) [26], CD166/activated leukocyte cell adhesion molecule (ALCAM) [19] and Thy-1/CD90 (a glycosylphosphatidylinositol-anchored glycoprotein) [22]. The hyaluronan receptor (CD44) and the adhesion molecules vascular cell adhesion molecule (VCAM)-1/CD106, and intercellular adhesion molecule (ICAM)-2/CD102 are also MSC markers [17,21,27-29]. The Notch-1 receptor with a role in maintaining stem cell pools and mediating stem cell fate is also considered a MSC marker [30,31]. MSC do not express markers of hematopoietic and endothelial cells such as CD11, CD14, CD31, CD33, CD34, CD45, and CD133 [17,32,33]. Despite the advances of identifying MSC from isolated cells, limited information concerning markers of such progenitor cells in the native tissue is available. However, recent studies on tissue-specific stem cell niches have been described and may be critical for identifying progenitors *in situ *[34].

Several joint tissues harbor multi-potential progenitors [35-37] including articular cartilage [10-12,38]. We previously identified a cell population in human adult articular cartilage that co-expressed the MSC markers CD105 and CD166 [10]. These cells did not express markers of differentiated chondrocytes and were capable of undergoing multilineage differentiation to chondrocytes, adipocytes, or osteoblasts. The superficial zone (SZ) of newborn bovine cartilage contains a subpopulation of cells that express Notch-1 and possess multilineage differentiation potential [38]. Similar observations were reported for equine and human articular cartilage [12,14,39,40]. An additional marker used to identify stem cells is based on the use of the Hoechst 33342 dye. By flow cytometry a cell population, termed 'side population' (SP) can be identified because it is not permanently stained by this dye since it expresses the multi-drug transporter ABCG2 (ATP-binding cassette, sub-family G) that removes the dye from the cell [41].

Towards establishing suitable means of identifying progenitor populations in articular cartilage, in this study, we determined the location and frequency of Notch-1, Stro-1, and VCAM-1 positive cells via immunohistochemistry and the frequency of SP cells using flow cytometry in normal and OA AHAC. We also examined the relation of these markers with the distribution of Sox9 because it is an important regulator of many chondrogenic genes [42].

## Materials and methods

### Cartilage procurement, grading, and processing

Normal and OA articular cartilage was obtained from tissue banks under approval by the Scripps human subjects committee. The knees were graded macroscopically (according to a modified Outerbridge scale where grade 1 represents intact surface, grade 2 minimal fibrillation, grade 3 overt fibrillation, and grade 4 full thickness defect [43]), and microscopically according to a modified Mankin scale with a score of less than three points being normal and a score of more than five to represent OA [44,45]. Some areas in OA joints did not exhibit surface fibrillations and were classified as 'OA non-fibrillated' versus fibrillated areas from OA joints that were classified as 'OA fibrillated'. Safranin O stained sections were used to determine whether all zones were represented.

### Cell isolation and culture

Cells were isolated from articular cartilage using collagenase as described [10]. The cells were cultured in Dulbecco's Modified Eagle's Medium (DMEM) (Mediatech, Inc., Manassas, VA, USA) supplemented with 10% calf serum (CS) and Penicillin-Streptomycin-Glutamine (Invitrogen, Carlsbad, CA, USA)). Cells were then cultured in monolayer culture at a seeding density of 50,000 cells/cm^2 ^for 24 hours (passage zero) or until confluence and split once (passage 1) at a seeding density of 10,000 cells/cm^2^.

### Immunohistochemistry

A total of 40 donors were used for immunohistochemistry (IHC) in this study. Seventeen donors were classified as normal (mean ± standard deviation age 38.8 ± 16.3 years; range 14 to 61 years; 6 females and 11 males) and 23 donors with OA (mean age of 64.7 ± 13.9 years; range 39 to 88 years; 11 females and 12 males). Cartilage from normal healthy and OA-affected donors (non-fibrillated OA and fibrillated OA) was embedded in paraffin. The total number of donors used for each marker and for each condition (normal, non-fibrillated OA, and fibrillated OA) is indicated in Table 1. Each paraffin block was sectioned (5 μm) and at least two sections from each donor were immunostained for detection of Notch-1 (1 μg/ml; Mouse IgG, Abcam, Cambridge, MA, USA), Stro-1 (0.5 μg/ml; Mouse IgM, R&D Systems, Minneapolis, MN, USA), VCAM-1/CD106 (1 μg/ml; Mouse IgG, Pharmingen/Becton Dickinson, San Jose, CA, USA), Sox9 (1 μg/ml; Rabbit IgG, Chemicon/Millipore, Temecula, CA, USA) and collagen type II (1 μg/ml; II-II6B3; Hybridoma Bank, University of Iowa, Iowa City, IA, USA). IHC was performed on sections of 5 μm in thickness using the Histostain-Plus kit (Zymed Laboratories, South San Francisco, CA, USA) following the manufacturer's instructions. Species-matched isotype controls (IgM; 0.5 μg/ml and IgG; 1 μg/ml) were used in combination and alone to monitor possible non-specific and cross-reactive staining. To show specificity of Sox9 staining, we used human fetal growth plates, as previously described by Aigner and colleagues [46].

**Table 1:** Percentage of positive immunostained Notch-1, Stro-1, VCAM-1, and Sox9 cells

**Molecule**	**Zone**	**Percentage positive (±SE)**			
		
		**Normal**	**OA Non-fibrillated**	**OA Fibrillated**	**OA clusters**
**Notch-1**	**Superficial**	71.5 ± 3.2†	57.7 ± 9.0*	84.2 ± 3.7†**#	83.6 ± 7.0 (1/5¶)
Normal (n = 8)^Δ^ OA NF (n = 5) OA Fib (n = 5)	**Middle**	34.8 ± 6.7	48.9 ± 6.6*	61.6 ± 7.4†**	80.9 ± 7.2 (6/40)
	**Deep**	29.1 ± 6.9	28.2 ± 10.6†	10.4 ± 5.6†**	68.68 ± 7.2 (2/11)
					**Mean: 78.5 ± 5.2**

**Stro-1**	**Superficial**	81.3 ± 5.9†	84.7 ± 4.4†	82.9 ± 2.2	90.5 ± 9.5 (2/17)
Normal (n = 9) OA NF (n = 8) OA Fib (n = 4)	**Middle**	51.8 ± 7.6	56.8 ± 5.4†	71.2 ± 12.7	75.1 ± 14.7 (4/31)
	**Deep**	38.3 ± 12.3	42.0 ± 8.2†	42.8 ± 20.6†	37.0 ± 9.15§ (2/11)
					**Mean: 69.4 ± 10.4**

**VCAM-1**	**Superficial**	84.1 ± 1.3†	82.5 ± 4.7†	90.8 ± 5.5†	88.6 ± 7.7 (2/14)
Normal (n = 4) OA NF (n = 6) OA Fib (n = 4)	**Middle**	41.0 ± 8.9†	65.3 ± 4.4†*	66.5 ± 6.3**	76.5 ± 8.9 (3/24)
	**Deep**	15.7 ± 5.5†	44.5 ± 6.8†*	60.7 ± 11.3**	61.1 ± 3.7 (3/19)
					**Mean: 75.4 ± 1.6**

**Sox9**	**Superficial**	68.5 ± 6.9†	48.5 ± 8.8*	69.0 ± 5.3#	81.8 ± 5.2 (3/27)
Normal (n = 8) OA NF (n = 8) OA Fib (n = 6)	**Middle**	48.4 ± 6.1	43.2 ± 7.1	43.0 ± 8.6	73.5 ± 4.9 (4/30)
	**Deep**	38.0 ± 9.3	39.5 ± 11.3	18.5 ± 11.7#	25.6 ± 11.4§ (4/27)
					**Mean: 71.6 ± 4.9**

Results show percentage positive cells for each zone in normal, non-fibrillated (NF), fibrillated (Fib) osteoarthritic (OA), and cells in OA cell clusters in human articular cartilage (number of donors).

† *P *< 0.05 between zone comparisons within each condition. * *P *< 0.05 between normal and non-fibrillated OA in the corresponding zone. ** *P *< 0.05 between normal and fibrillated OA in the corresponding zone. # *P *< 0.05 between non-fibrillated OA and fibrillated OA in the corresponding zone. ¶ (number of donors assessed/total number of clusters counted). **§ ***P *< 0.05 between clusters in each zone within each condition. ^Δ ^Number of donors stained for each marker and condition.

SE = standard error; VCAM-1 = vascular cell adhesion molecule-1.

### Quantification of immunostaining patterns throughout adult human articular cartilage

**Figure 1:**
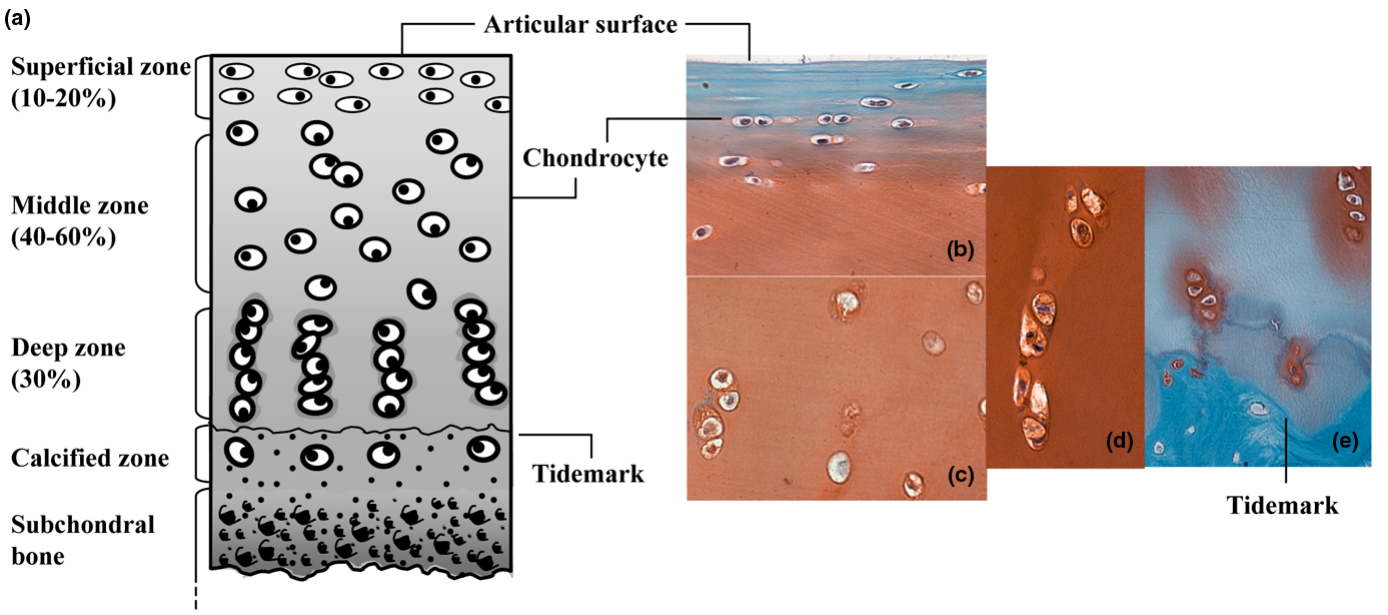
Overview of cartilage structure and zonal architecture and representative Safranin O micrographs of cells in each zone. **(a) **Adapted from Tyyni and Karlsson [65]. Identification of each zone was based on previously reported characteristics that comprise cell shape, morphology, orientation, and pericellular matrix (PM) deposition [47]. Superficial zone (SZ) cells are small, elongated in shape, parallel relative to the surface, and lack an extensive PM. These cells predominate the first 50 μm. The middle zone (MZ) is distinguishable by rounded cells that do not exhibit an organized orientation relative to the surface, are within ECM rich in proteoglycans and show presence of PM. Deep zone (DZ) cells were identified by an extensive PM deposition with chondrons in groups of three or more cells arranged in columns perpendicular to the surface. Safranin O staining of the **(b) **SZ and upper MZ, **(c) **MZ, **(d) **DZ chondrocytes and **(e) **DZ and calcified zone.

Assessment of positive signal localizations throughout each cartilage zone included systematic counting of positive and negative cells in a 50 × 50 μm grid (40× field), starting from the cartilage surface, down through the full thickness tissue specimen. This was repeated five times for each section (minimum of two sections per donor). The identification of each zone was based on previously reported characteristics [47] (Figure 1). The frequency of positive signals was calculated for each zone. To assess staining frequencies in OA cartilage sections with extensive surface fibrillations, where the SZ was absent or would not be recognizable, assessment proceeded from the deep zone (DZ) up to the fibrillated surface. In extensively fibrillated samples, the fibrillated surfaces were considered middle zone (MZ). We also examined sections that appeared normal with intact surface from OA joints (non-fibrillated OA).

### Flow cytometry

Primary isolated human articular chondrocytes were detached from culture flasks after 24 hours of culture following isolation from cartilage or after the first passage (approximately three weeks in culture) using Accutase (Innovative Cell Technologies, Inc. San Diego, CA, USA), washed in PBS, resuspended in PBS/BSA (1%), and divided into 1.5 ml Eppendorf tubes (1 × 10^3^). The cells were stained with 4 μg/ml CD44 (4 μg/ml; Diaclone/Tepnel Lifecodes Corp., Stamford, CT, USA), CD105 (4 μg/ml; Mouse IgG, Ancell, Bayport, MN, USA), CD90 (4 μg/ml; Mouse IgG, Serotec, Kidlington, Oxford, UK), CD166 (4 μg/ml; Mouse IgG, Ancell, Bayport, MN, USA), Stro-1 (10 μg/ml; Mouse IgM, R&D Systems, Minneapolis, MN, USA), and Notch-1 (L18, 4 μg/ml; Goat Polyclonal, Santa Cruz Biotechnology, Inc., Santa Cruz, CA, USA). Species-matched isotype controls were used at the same concentrations. All antibody incubations (primary and secondary) were performed on ice for 30 minutes each. The cells were subjected to fluorescence-activated cell sorter (FACS) analysis using a Becton Dickinson FACScan and Cell Quest software (Becton Dickinson, San Jose, CA, USA). The extent of positive staining was calculated as a percentage in comparison with the isotype control staining, set at the 1% level. Signals less than 1% were considered negative.

### Quantitative real-time PCR

Total RNA was isolated from monolayer or pellet cultures using Trizol (Invitrogen, Carlsbad, CA, USA). cDNA was produced using Ready-to-go You-Prime First-Strand Beads (GE Healthcare Life Sciences, Uppsala, Sweden) with total RNA 1 μg and oligo (dT)18 primers. Quantitative real-time RT-PCR (qPCR) was performed using TaqMan Gene Expression Assay probe for *ABCG2 *(Hs00194979_m1), *Sox9 *(Hs00165814_m1),* Col2a1 IIA *(Hs00156568_m1), *Col2a1 IIB *(Hs01064869_m1), *Aggecan *(Hs00202971_m1), *Col1a1 *(Hs00164004_m1), *Col10a1 *(Hs00166657_m1), *Runx2 *(Hs00298328_s1), *Osterix *(Hs00541729_m1), *Osteocalcin *(Hs01587814_g1), *Adiponectin *(Hs02564413_S1), and *GAPDH *(Hs99999905_m1) (All Applied Biosystems, Foster City, CA, USA). Relative expression was calculated using the ΔΔC_t _values and results were expressed as 2^-ΔΔCt^. *GAPDH *was used as an internal control to normalize differences in each sample.

### Side population isolation and culture

Human articular chondrocytes in first passage monolayer culture were incubated in Hoechst dye 33342 (4 μg/ml) at 37°C for 90 minutes, washed in ice cold Hank's balanced salt solution and maintained on ice. Propidium iodide (2 μg/ml) was added just prior to sorting to exclude dead cells. The FACSVantage SE flow cytometer (Becton Dickinson, San Jose, CA, USA) was used to determine the frequency of Hoechst negative cells (SP cells) and to isolate SP and non-SP (NSP) chondrocytes. Sorted cells were placed in culture and expanded in DMEM supplemented with 10% CS and Penicillin-Streptomycin-Glutamine. SP and NSP cells were cultured for six passages (>25 cell doublings) to achieve adequate numbers for the differentiation assays.

### Chondrogenesis assay

Cells from each population (SP and NSP) were placed into pellet cultures (0.5 × 10^6^/pellet) in Insulin, Tranferrin, Selenium (ITS+) serum free medium (Sigma, St. Louis, MO, USA) supplemented with TGFβ1 (10 ng/ml) for two weeks. Pellets were processed for histology (Safranin O staining) and RT-PCR analyses. Total RNA was extracted using RNA easy kit (Qiagen, Valencia, CA, USA) and cDNA was generated using the ready-to-go-first-strand beads kit (GE Healthcare Life Sciences, Uppsala, Sweden). Expression levels of *ABCG2*, *Col1a1*, *Col2a1 IIA*, *Col2a1 IIB*, *Col10a1*, *Sox9*, and *aggrecan *(normalized to *GAPDH*) were assessed via qPCR.

### Osteogenesis assay

Osteogenic differentiation was also analyzed in monolayer cultures using established medium supplements [48,49]. Cells were seeded in 24-well plates (1 × 10^3 ^each well) in DMEM plus 10% CS, 10 nM dexamethasone, 10 mM β-glycerophosphate, and 0.1 mM L-ascorbic acid-2-phosphate (Sigma, St. Louis, MO, USA) and cultured for three weeks. Medium was changed twice weekly. Negative control wells were maintained in DMEM supplemented with 10% CS for the duration of the assay. Cells were harvested for RNA extraction and qPCR to examine the expression of *Runx2*, *Osterix*, *Osteocalcin*, and *Col1a1*.

### Adipogenesis assay

Adipogenesis of SP and NSP cells was induced in monolayer cultures employing induction and maintenance media as previously described by Pittenger and colleagues [17]. Briefly, 1 × 10^3 ^cells were seeded in 24-well plates and cultured with DMEM supplemented with 10% CS until confluent. These cells were exposed to the induction medium consisting of 10 μg/ml insulin, 1 μM dexamethasone, 500 μM 3-isobutyl-1-methyl xanthine, 100 μM indomethacin (Sigma, St. Louis, MO, USA) for 72 hours. The medium was replaced with maintenance medium, 10 μg/ml insulin in DMEM, and 10% CS, and culture was continued for 24 hours. This 96-hour treatment cycle was repeated four more times, followed by culture for an additional week in adipogenic maintenance medium. Negative control wells were maintained in DMEM supplemented with 10% CS for the duration of the assay. The cells were harvested for qPCR analysis of *Adiponectin*.

### Statistical analysis

Comparisons between each zone, between normal and non-fibrillated OA and between non-fibrillated OA and fibrillated OA tissue were made via one-way analysis of variance (ANOVA) followed by student's t-tests (Microsoft Excel, version 11.3.5, Redmond, WA, USA). *P *values less than 0.05 were considered significant.

## Results

### Distribution of Notch-1, Stro-1, VCAM-1, and Sox9 in normal adult human articular cartilage

**Figure 2:**
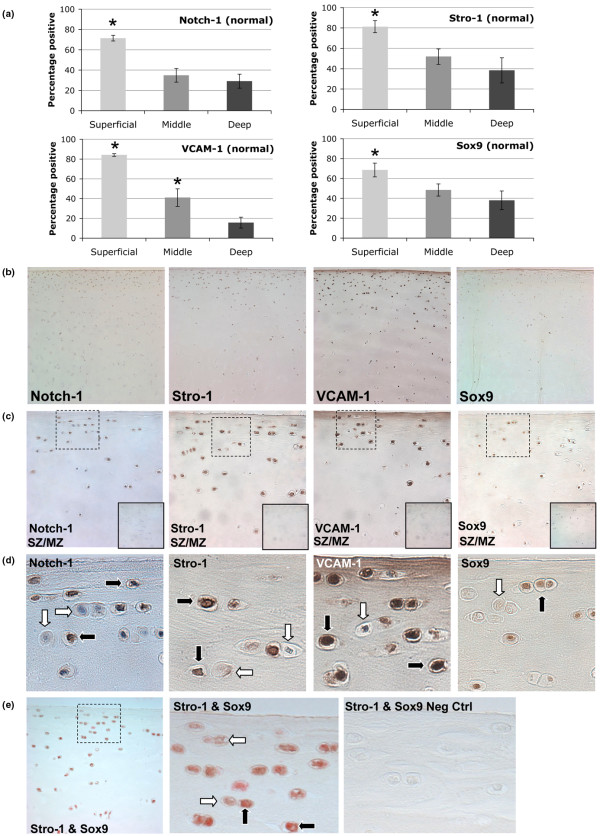
Distribution of Notch-1, Stro-1, VCAM-1, and Sox9 in normal human adult articular cartilage. **(a) **Percentage positive signal for the superficial zone (SZ), middle zone (MZ), and deep zone (DZ). *P < 0.05. **(b) **Representative images (10×) for Notch-1, Stro-1, VCAM-1, and Sox9 showing greater staining frequency in the SZ and upper MZ. **(c) **Images depicting SZ and upper MZ (40×). Solid inset (bottom right) indicates negative controls. Dotted line box outlines SZ images presented in **(d) **showing a mix of cells that are positive (black arrow) or negative (white arrow) for each immunostain. **(e) **Stro-1 (brown) and Sox9 (red) double staining with some cells single Stro-1 positive (white arrow) or Stro-1/Sox9 double positive (black arrow) (40×).

A surprisingly high number of cells stained positive for the MSC markers Stro-1, VCAM-1, and Notch-1 in normal human articular cartilage. On average, combining all zones, over 45% of cells were positive (Figure 2 and Table 1).

There were significant zonal variations in marker expression. Over 70% of cells in the SZ were Notch-1 positive (Table 1 and Figure 2a), but significantly less were positive in the MZ (35%) and DZ (29%). The SZ also contained significantly higher numbers of Stro-1 (81%) and VCAM-1 (84%) positive cells compared with MZ and DZ cells (Table 1). Representative images are shown in Figure 2.

Chondrocyte differentiation and the expression of cartilage matrix genes are in part regulated by Sox transcription factors [42]. Sox9 was detected in all zones in approximately 50% of all chondrocytes (Table 1 and Figure 2). A significantly higher percentage of cells in the SZ (69%) were positive for Sox9 compared with the other two zones (Table 1 and Figure 2a). The isotype and species matched controls indicate that all staining patterns observed in this study were specific (Figure 2c). Moreover, cells that are in close proximity or adjacent to each other can be positive or negative (Figure 2d). Sox9 staining specificity was confirmed using human fetal growth plate cartilage, showing that the majority of cells in the surface, resting, and proliferation zones positive and mostly negative in the hypertrophic zone (data not shown). Double staining of normal cartilage for Stro-1 and Sox9 showed that a majority of cells in each zone were double positive, although some cells, particularly in the SZ, can be detected as Stro-1 positive only (Figure 2e).

### Stem cell markers in human OA articular cartilage

**Figure 3:**
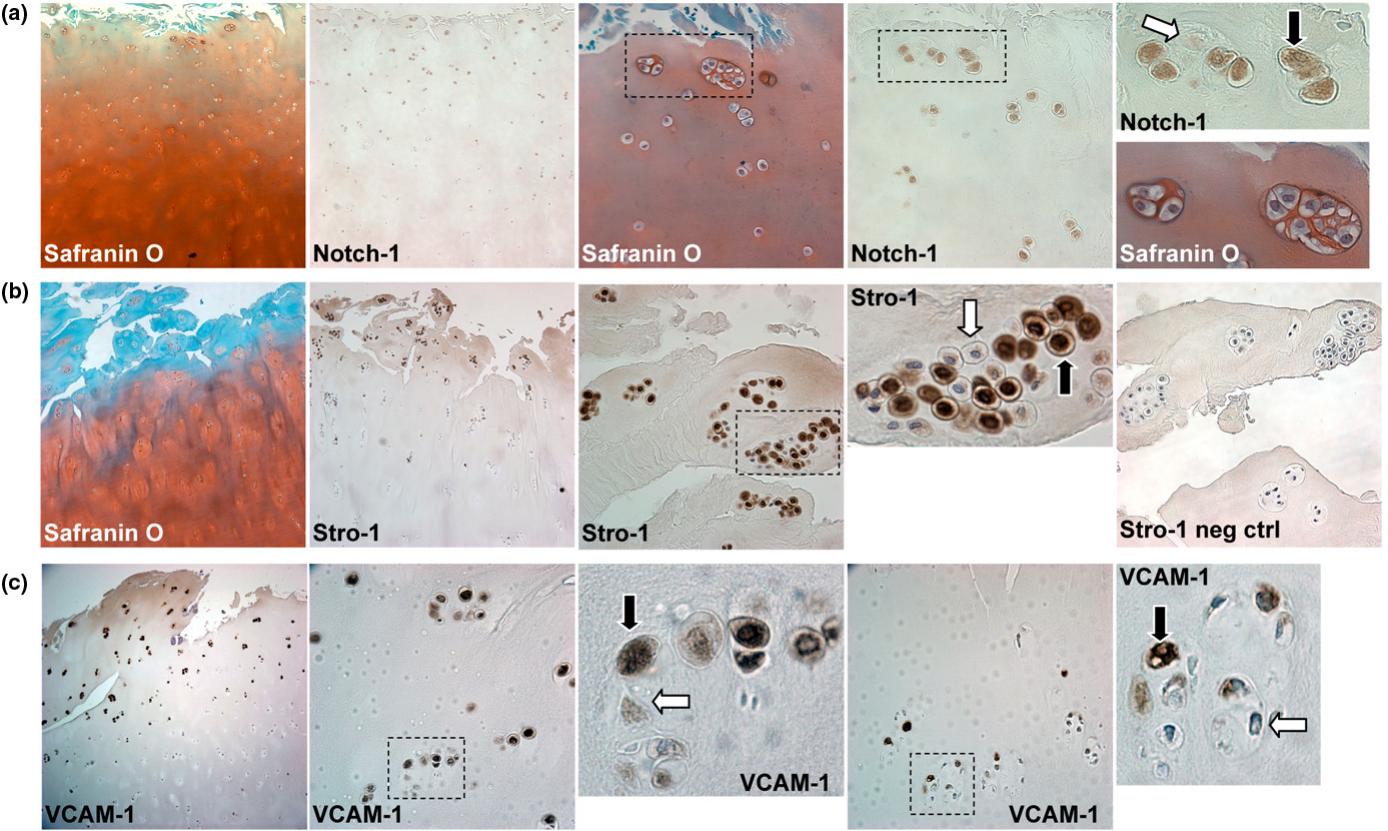
Stem cell markers in human osteoarthritis (OA) articular cartilage. **(a) **Safranin O and Notch-1 staining in clusters (10× and 40×). **(b) **Safranin O and Stro-1 staining of OA cartilage sections (10× and 40×). **(c) **OA cartilage sections immunostained for VCAM-1 (10× and 40×). Positive staining indicated by black arrows and negative with white arrows.

In the SZ of non-fibrillated OA cartilage there was a significant reduction of Notch-1-positive cells as compared with normal cartilage (71.5% in normal to 57.7% in OA; Table 1). By contrast in fibrillated OA samples, where we could still identify the SZ, Notch-1 frequency significantly increased to an average of 84.2%, relative to normal (71.5%). The increased frequency of Notch-1 in fibrillated cartilage was a reflection of the multiple cell clusters present in these tissues (Figure 3).

In the MZ, Notch-1 staining increased in non-fibrillated OA cartilage to 48.9% and further in fibrillated cartilage to over 60% (Table 1). In the DZ of non-fibrillated OA cartilage there were significantly less Notch-1-positive cells (28.2%) compared with normal cells and this value decreased further to 10.4% in the DZ of fibrillated OA tissues (Table 1).

Stro-1 staining was not significantly different in the SZ of normal versus OA samples. In the MZ of OA-affected cartilage there was a trend towards higher Stro-1 staining as compared with normal.

VCAM-1 staining was similar in the SZ of normal and OA cartilage. A significant increase in VCAM-1 staining frequency was detected in the MZ and DZ of OA-affected tissues (Table 1). All three markers showed decreased expression from the SZ to the MZ and DZ of OA tissues.

The frequency of Sox9-positive cells was significantly reduced in the SZ of non-fibrillated OA cartilage (49%) compared with the SZ of normal cartilage (69%). No significant alteration in Sox9 frequency was seen in the MZ and DZ of non-fibrillated OA cartilage compared with normal. The number of Sox9-positive cells in MZ remained unchanged in the fibrillated cartilage, yet a significant increase was noted in the SZ of fibrillated tissue to levels similar to those in normal SZ cartilage. In comparison with the DZ of non-fibrillated cartilage (40%), Sox9 frequencies significantly fell to 19% in the DZ of fibrillated cartilage.

In summary, the SZ of non-fibrillated OA cartilage showed reduced Notch-1 and Sox9 staining frequency. Yet, the MZ showed increased frequency of Notch-1 and VCAM-1 in non-fibrillated and fibrillated OA tissue. Finally, the DZ had decreased levels of both Notch-1 and Sox9 staining in fibrillated OA tissue, although the number of VCAM-1-positive cells increased.

### Cell clusters in OA cartilage express progenitor markers

**Figure 4:**
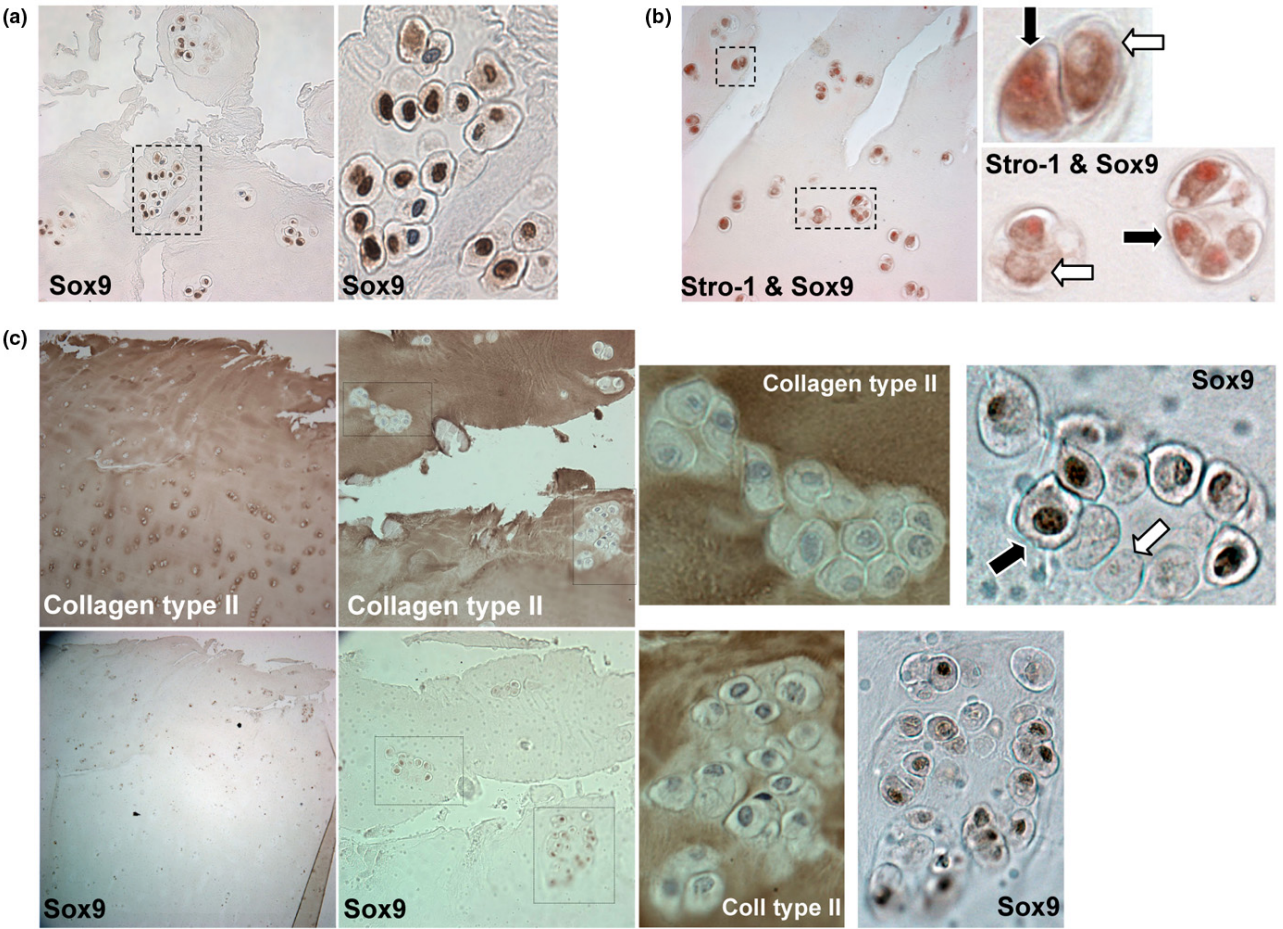
Cell cluster staining for Sox9, Stro-1, and collagen type II. **(a) **Cells in clusters can be negative (white arrow) for Sox9 or show cytoplasmic and/or nuclear staining (black arrow) (10×). **(b) **Double staining with Stro-1 (brown) and Sox9 (red) indicate cells that are single (white arrow) or double positive (black arrow) (40×). **(c) **Collagen type II and Sox9 immunostaining of osteoarthritis (OA) cartilage. Clusters are surrounded by collagen type II matrix and not all cells in these clusters are Sox9 positive (black arrow positive; white arrow negative) (10× and 40×).

The number of cell clusters was increased in fibrillated OA cartilage (Figure 3). A majority of cells in clusters (69 to 79%) were positive for Notch-1, Stro-1, VCAM-1, and Sox9 (Table 1). Clusters located in the DZ had significantly reduced frequencies of Stro-1 and Sox9-positive cells (Table 1). Not all cells in clusters were positive for Notch-1, Stro-1, VCAM-1, or Sox9 (Figure 3). Moreover, Sox9 staining patterns were mainly nuclear in normal cartilage (Figure 2), but Sox9 staining in OA clusters was present in both the cytoplasm and nucleus, or even exclusively in the cytoplasm (Figure 4a). Stro-1 and Sox9 double-staining images (Figure 4b) indicate that cells within clusters can be double positive for Stro-1 and Sox9 (black arrows) or single positive for Stro-1 (white arrows). Cells within clusters are surrounded by an ECM rich in type II collagen, but not all cells stained positive for Sox9 (Figure 4c).

### Stem cell marker expression in isolated cartilage cells

To extend the IHC results, cells were isolated and analyzed in first passage by flow cytometry. Contrasting the high frequency of Notch-1 and Stro-1-positive cells as detected by IHC in cartilage, flow cytometry showed much lower expression levels of these markers (Normal: n = 4; 37.8 ± 5.9 years old; OA: n = 4. 61.5 ± 5.7 years old). Notch-1-positive cells in normal cartilage cells were 2.4% and 3.5% in OA, while Stro-1 levels were 5.4% and 7.6% in normal and OA, respectively. To clarify the discrepancy between IHC and FACS observations, we stained cells 24 hours after enzymatic isolation in 10 donors (ages and gender indicated in Table 2). Stro-1 levels in cells cultured for only for 24 hours were 25.6 ± 5.2% (Table 2), but this dropped to below 10% by seven days (data not shown). Notch-1 levels were lower at 4.7 ± 1.2% (Table 2). No significant shift in Notch-1 or Stro-1 expression levels were detected between 24-hour cultured normal and OA cells. Of the other progenitor markers investigated at first passage, 48.7 ± 11.4% of cells from OA cartilage (n = 4 donors) were positive for CD166 as opposed to only 8.4 ± 4.8% in cells from normal cartilage (*P *< 0.05; n = 4 donors). There was a trend towards increased CD105 levels in OA cells (normal: 57.3 ± 21.2%; OA: 80.1 ± 8.8%). CD44 and CD90 surface molecule expression levels did not significantly differ between normal and OA cells. These results from isolated cells show much lower stem cell marker expression as compared with cartilage tissue. This may be the result of cell loss during isolation or down regulation of the markers during cell isolation and subsequent culture.

**Table 2:** Flow cytometric analysis of human chondrocytes derived from normal and OA-affected articular cartilage, cultured in monolayer for 24 hours (n = 10) and stained for Stro-1 and Notch-1

			**Percentage positive**	
			
	**Age and gender**	**OA grade†**	**Stro-1**	**Notch-1**
Donor 1	53 male	1	25.1	6.1
Donor 2	17 female	1	9.5	4.3
Donor 3	65 male	1 to 2	nd*	14.0
Donor 4	61 male	2	26.5	2.8
Donor 5	30 male	2	54.6	1.6
Donor 6	56 female	2	29.1	3.4
Donor 7	65 female	2	nd	4.2
Donor 8	64 male	2 to 3	22.7	3.1
Donor 9	59 male	3 to 4	31.0	nd
Donor 10	64 male	3 to 4	5.9	2.5
				
		**Mean ± SE**	**25.6 ± 5.2**	**4.7 ± 1.2**

† Grade 1 represents intact surface, Grade 2 minimal fibrillation, Grade 3 overt fibrillation and Grade 4 full thickness defect. *nd = not determined; OA = osteoarthritis; SE = standard error.

### Side population

**Figure 5:**
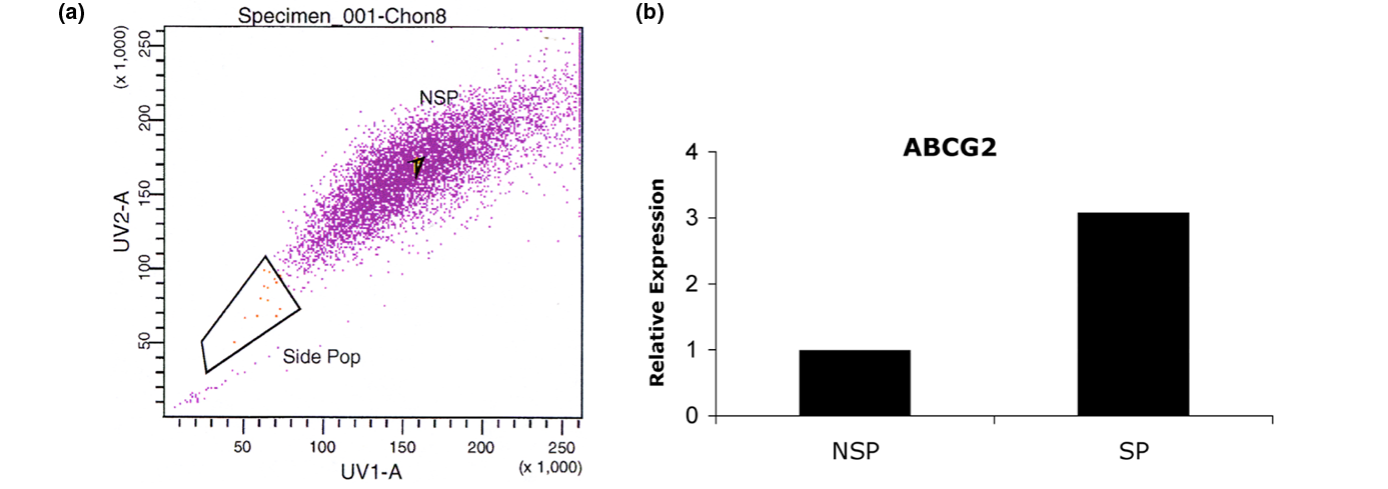
Side population in normal cartilage. **(a) **FACS image of the gated side population (SP) and non-SP (NSP) cells isolated via cell sorting. **(b) **Expression level of ABCG2 in SP and NSP cells. The three-fold higher expression of ABCG2 indicates successful isolation of the cartilage SP.

**Figure 6:**
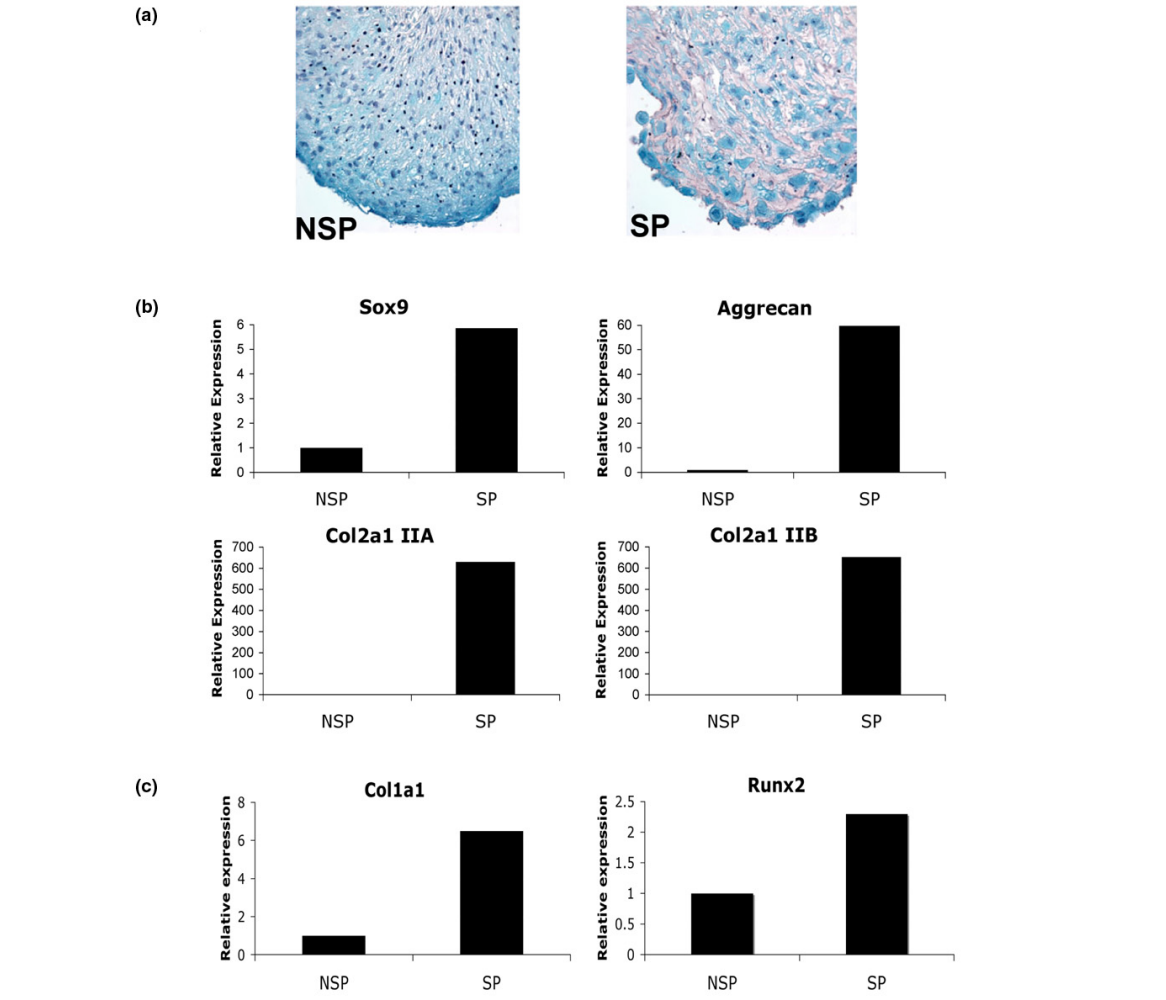
Multilineage potential of the side population (SP) derived from normal human articular cartilage. **(a) **Safranin O staining of 14-day SP and non-SP (NSP) pellet cultures (magnification 40×). **(b) **Gene expression analysis of 14-day pellet cultures relative to NSP cells. Higher *Sox9*, *Aggrecan*, and both *Col2a1 IIA *and *Col2a1 IIB *expression in SP cells. **(c) **SP cultured in pro-osteogenic medium for three weeks show higher levels of *Col1a1 *and *Runx2 *gene expression relative to NSP cells.

The overall frequency of SP cells in first passage monolayer cells from normal articular cartilage (n = 4; 42.3 ± 12.1 years) was 0.15 ± 0.06% and 0.13 ± 0.06% in OA (n = 3; 51.0 ± 8.5 years old). A three-fold higher level of transmembrane transporter protein ABCG2 in isolated SP cells, compared with NSP, confirmed successful collection of the SP by flow cytometry (Figure 5). SP cells were found to have higher chondrogenic potential compared with NSP as seen by Safranin O staining (Figure 6a) and gene expression (*Col2a1 IIA, IIB*, *Sox9*, and *aggrecan*) (Figure 6b). The expression of *Runx2 *and high expression of *Col1a1 *in SP cells cultured in pro-osteogenic conditions revealed osteogenic differentiation potential (Figure 6c). *Osteocalcin *and *Osterix *were not detected. No evidence of adipogenic differentiation was observed (data not shown).

## Discussion

The current study was designed to determine the localization of cells expressing putative progenitor markers in normal and OA human articular cartilage. The three selected candidate markers Notch-1, Stro-1, and VCAM-1 have been widely used to identify bone marrow MSC [23,25,28-31]. Staining patterns for the three markers in normal human articular cartilage were similar with significantly higher staining frequency in the SZ as compared with the MZ and DZ. This is consistent with observations from other laboratories using the same or other stem cell markers [12,38,40]. Using IHC we observed a surprisingly high frequency of cells expressing Notch-1, Stro-1, and VCAM-1 throughout normal human articular cartilage. Using flow cytometry as an alternative method to detect Notch-1 and Stro-1 we observed lower levels of positive cells as compared with IHC. Furthermore, although the percentage of Notch-1 and Stro-1-positive cells was similar by IHC, the flow cytometry results showed much higher expression of Stro-1 as compared with Notch-1. As we demonstrated specificity of the IHC signals, these results suggest that profound changes in the expression of these markers occur upon cell isolation and that the patterns of change are different for each marker. This change could either be the result of a downregulation of protein expression in monolayer culture, indicate a sensitivity to exposure to collagenase digestion, previously demonstrated for numerous surface molecules on human articular chondrocytes [50] or be because of preferential loss of cells expressing these markers during the isolation process. Enzymatic digestion of cartilage recovers less than 22% of the total number of cells present in the original tissue [51], indicating that certain subpopulations such as those expressing progenitor markers may be lost.

Given the unexpected high levels of Notch-1, Stro-1, and VCAM-1-positive cells in cartilage, we applied an additional means of identifying stem cells. The Hoechst dye 33342, which defines the so-called SP, was used with freshly isolated cells from human articular cartilage and on flow cytometry we observed that the SP represented only 0.1% of the cells. This frequency is similar to that reported for young bovine cartilage [52]. However, this is vastly different from the frequency of Notch-1, Stro-1, and VCAM-1-positive cells. The Hoechst dye thus appears to be a more appropriate stem cell marker.

In the present study we did not examine whether the cells expressing Notch-1, Stro-1, or VCAM-1 had multilineage differentiation capacity. Our previous study [10] and Dowthwaite and colleagues [38] demonstrated that cartilage cells expressing CD105/CD166 or Notch-1 do indeed have stem cell activities. However, Karlsson and colleagues [53] recently demonstrated that Notch-1 is not a progenitor marker in cartilage. To reconcile these observations in reflection to this current data set, it is most plausible that a subpopulation of these identified progenitor-positive cells is multi-potent, which is represented by the SP. Further surface molecule characterization of the cartilage SP is required.

Based on the differences in the frequency of Notch-1, Stro-1, or VCAM-1-positive cells versus SP cells, these represent very different cell populations. We propose that the observed high frequency of progenitors in cartilage is a reflection of multiple functions that these progenitor molecules have in the native tissue such as controlling cell fate, proliferation, and apoptosis [30,54,55]. On the other hand, cartilage may contain a very high proportion of progenitor cells due to its avascular quality. Frequency of Notch-1-positive cells among different human tissues ranges from 0 to more than 60% [56]. The concentration of stem cells in the SZ and on the surface of developing human cartilage is also consistent with a recent report [57] showing that during postnatal development of rabbit knee joints, the SZ contains stem cells that supply a rapidly dividing, transit-amplifying daughter-cell pool. Following cessation of growth and attainment of joint maturation the stem cell pool in the SZ may provide a reservoir for replenishing cells in the cartilage surface that is the site of biomechanical load and wear. Based on the present results this cellular organization appears also present and maintained in mature human articular cartilage.

This study is the first to analyze changes in the distribution of stem cell markers in OA affected human articular cartilage. High Stro-1 protein expression levels have been observed in OA synovium cell clusters [37] and the soluble form of VCAM-1 has been implicated in rheumatoid arthritis and OA [58,59]. We have previously reported increased expression of Notch-1 in OA cartilage [14] and a recent study indicates that Notch-1-positive cells and its signaling components, Jagged1 and Hes5, are upregulated in OA and mediate cell proliferation [40]. The reduction in both Notch-1 and Sox9 in the SZ non-fibrillated OA cartilage is notable because this implies a reduction in progenitor cells and probably normal cartilage ECM production, respectively. This shift may be a consequence of aging and such cell depletion may be an important initiator or a predisposing factor leading to OA development. We have recently demonstrated co-ordination between Notch-1 and Sox9 signaling to either inhibit or promote chondrogenesis [60]. Imbalance between these pathways may be an inherent feature of OA and a possible therapeutic target.

OA cartilage is characterized by cell cluster formation and abnormal cell differentiation processes with renewed expression of cartilage development related extracellular matrix components [61-63]. Genes attributed to dedifferentiated (collagen types I and III, fibronectin) and hypertrophic chondrocytes (collagen type X) are also detected in OA clusters [6,61]. Based on the present observations, the cells that compose these clusters are likely to be a result of proliferating chondroprogenitors. Aigner and colleagues [64] indicated that MZ cells are principally activated in OA tissue and these cells express type IIA procollagen, indicative of the chondroprogenitor phenotype, which is in agreement with our current observations of increased progenitor markers in the same area of OA cartilage. Fukui and colleagues [61] showed the most profound phenotypic shift as indicated by the expression of type II collagen and fibronectin in OA fibrillated areas where clusters are prominent. Understanding the basis of such aberrant chondrocyte responses and whether resident progenitor cells are involved will be vital for the development of therapies and diagnostic markers to control and prevent OA progression.

Results from the marker staining patterns in OA cartilage show several changes as compared with normal tissue. However, the type of change is also specific for each marker. For example, there is a marked decrease in Notch-1 in the DZ of fibrillated OA cartilage but VCAM-1 is increased by four-fold. These divergent changes further suggest that the selected cell surface receptors are at least in part independently regulated as part of the cell activation process in OA and do not represent suitable stem cell markers in cartilage.

## Conclusions

Although the progenitor cell markers analyzed in this study can not be considered alone as representative indicators of stem cells within human articular cartilage, the increased presence of such molecules in OA tissue, in particular in cell clusters, further implicates their involvement in the abnormal matrix remodeling process. In particular, Notch signaling is known to modulate cell proliferation, apoptosis and differentiation, which may represent a target for modulating OA disease progression.

## Abbreviations

ABCG2: ATP-binding cassette, sub-family G; AHAC: adult human articular cartilage; ALCAM: activated leukocyte cell adhesion molecule; ANOVA: analysis of variance; BM-MSC: bone marrow-derived mesenchymal stem cell; BSA: bovine serum albumin; DMEM: Dulbecco's Modified Eagle's Medium; DZ: deep zone; FACS: fluorescence-activated cell sorter; ICAM-2: intercellular adhesion molecule-2; IHC: immunohistochemistry; MSC: mesenchymal stem cell; MZ: middle zone; NSP: non-side population; OA: osteoarthritis; PBS: phosphate buffered saline; RT-PCR: reverse-transcriptase polymerase chain reaction; SP: side population; SZ: superficial zone; TGFβ1: Transforming growth factor beta-1; VCAM-1: vascular cell adhesion molecule-1.

## Competing interests

The authors declare that they have no competing interests.

## Authors' contributions

SPG participated in study conception and design, acquisition of data (immunohistochemistry, histomorphometry, isolation of side population (SP) and culture, FACS analysis), analysis and interpretation of data, and drafting the manuscript. SM participated in study conception and design, acquisition of data (isolation of SP and gene expression analysis), and analysis and interpretation of data. HA participated in analysis and interpretation of data. DDL participated in analysis and interpretation of data, and drafting the manuscript. MKL participated in study conception and design, analysis and interpretation of data, and drafting the manuscript. All authors read and approved the final manuscript.
